# The *Aeromonas salmonicida* Lipopolysaccharide Core from Different Subspecies: The Unusual subsp. *pectinolytica*

**DOI:** 10.3389/fmicb.2016.00125

**Published:** 2016-02-11

**Authors:** Susana Merino, Juan M. Tomás

**Affiliations:** Departamento de Microbiología, Facultad de Biología, Universidad de BarcelonaBarcelona, Spain

**Keywords:** *Aeromonas salmonicida*, typical and atypical, lipopolysaccharide core, inner and outer core chemical structure, genomics

## Abstract

Initial hydridization tests using *Aeromonas salmonicida* typical and atypical strains showed the possibility of different lipopolysaccharide (LPS) outer cores among these strains. By chemical structural analysis, LPS-core SDS-PAGE gel migration, and functional and comparative genomics we demonstrated that typical *A. salmonicida* (subsp. *salmonicida*) strains and atypical subsp. *masoucida* and probably *smithia* strains showed the same LPS outer core. *A. salmonicida* subsp. *achromogenes* strains show a similar LPS outer core but lack one of the most external residues (a galactose linked α1-6 to heptose), not affecting the O-antigen LPS linkage. *A. salmonicida* subsp. *pectinolytica* strains show a rather changed LPS outer core, which is identical to the LPS outer core from the majority of the *A. hydrophila* strains studied by genomic analyses. The LPS inner core in all tested *A. salmonicida* strains, typical and atypical, is well-conserved. Furthermore, the LPS inner core seems to be conserved in all the *Aeromonas* (psychrophilic or mesophilic) strains studied by genomic analyses.

## Introduction

The smooth lipopolysaccharide (LPS) in Gram-negative bacteria consists of large amphiphilic molecules with a hydrophilic polysaccharide and a hydrophobic highly conserved lipid component covalently bound. This lipid, named lipid A, is the bioactive endotoxin subunit. The polysaccharide section is mainly formed by two parts: one more internal and conserved, the core region, and one more external and highly variable, the O-specific chain, named also O-antigen for its immunogenic properties. Smooth LPS molecules show both polysaccharide parts, while rough LPS molecules only the completed or truncated LPS core. The lipid A, LPS-core, and O-antigen LPS have been differentiated and formally classified by their chemical structure, degree of conservation, biosynthetic pathways and genetic determination (see general review Aquilini and Tomás, 2015).

The LPS-core is also subdivided in two regions: inner and outer core. Within a genus or family, the structure of the inner core tends to be well-conserved, and typically consists of unusual sugars, particularly 3-deoxy-D-manno-oct-2-ulosonic acid (Kdo) and heptoses (Hep; Holst, 2002). The outer core shows more structural diversity, is characterized by more common hexose sugars such as glucose (Glc), galactose (Gal), *N*-acetyl galactosamine (GalNAc) and *N*-acetyl glucosamine (GlcNAc), and is more variable than the inner core (Holst, 2007).

*Aeromonas salmonicida* subsp. *salmonicida* is the typical etiological agent responsible of a systemic fish disease named furunculosis, which converts this bacterium into an important pathogen (Scott, 1968). This pathogen has been subjected to considerable investigation because of its enormous importance in the farmed fish industry. Its major virulence factor is an S-layer (named A-layer), which principally consists of a unique two-dimensional crystalline tetragonal protein (A-protein with a molecular weight of 49 KDa) array (Ishiguro et al., 1981), tethered to the cell by LPS (Belland and Trust, 1985). Some studies chemically characterized the LPS O-antigen polysaccharide and the core oligosaccharide region structures from *A. salmonicida* strain SJ-15 (Shaw et al., 1983, 1992). Furthermore, recently the functional genomics of the LPS O-antigen and A-layer from typical and atypical strains were described (Merino et al., 2015), and concluded that all the *A. salmonicida* typical and atypical strains, other than *A. salmonicida* subsp. *pectinolytica* strains, shared the same LPS O-antigen and presence of A-layer. *A. salmonicida* subsp. *pectinolytica* strains present another type of O-antigen LPS and lack one of the major virulence factors, the A-layer (Merino et al., 2015).

Other studies reported the chemical structure of *A. salmonicida* subsp. *salmonicida* LPS core oligosaccharide region from strains A449 and 80204-1 (Wang et al., 2006). Also, we were able to establish the *A. salmonicida* subsp. *salmonicida* strain A450 genomics and proteomics for the LPS-core (*waa*_salmo_), which is distributed in three different chromosomal gene locations (Jimenez et al., 2009).

Regions 2 and 3 showed identical genes between *A. salmonicida* subsp. *salmonicida* strain A450 and A449, and also with *A. hydrophila* AH-3 (Jimenez et al., 2008, 2009). *A. salmonicida* subsp. *salmonicida* strains A450 and A449 region 1 showed seven identical genes, three of them identical to *A. hydrophila* AH-3, three of them similar but not identical to *A. hydrophila* AH-3, and one of them totally different that seems to be specific for *A. salmonicida* subsp. *salmonicida* (Jimenez et al., 2008, 2009).

Currently, there are five accepted subspecies of *Aeromonas salmonicida*: *A. salmonicida* subsp. *salmonicida* (known as typical), *masoucida*, *achromogenes*, *pectinolytica*, and *smithia* (Austin and Austin, 2007). Atypical *A. salmonicida* include subsp. *smithia*, subsp. *masoucida*, subsp. *achromogenes* and subsp. *pectinolytica* which, with the exception of *A. salmonicida* subsp. *pectinolytica*, are found as pathogens in a wide variety of fish species. In this work we studied the three chromosomal regions encoding the LPS core biosynthesis by comparative analysis of published complete genomes of different strains of *A. salmonicida* subspecies, as well as the chemical LPS core structure for subspecies *masoucida*, *achromogenes*, and *pectinolytica* strains.

## Materials and Methods

### Bacterial Strains, Plasmids, and Growth Conditions

Bacterial strains, and plasmids used in this study are listed in **Table 1**. *Aeromonas* strains were routinely grown on tryptic soy broth (TSB) or tryptic soy agar (TSA) at 20°C (*A. salmonicida*) and 30°C (*A. hydrophila*). *Escherichia coli* strains were grown in Luria-Bertani Miller broth and on the same medium with agar at 37°C. Kanamycin (50 μg ml^-1^), ampicillin (100 μg ml^-1^), rifampicin (100 μg ml^-1^), nalidixic acid (20 μg ml^-1^), or gentamicin (20 μg ml^-1^) were added to the different media when required.

**Table 1:** Bacterial strains and plasmids used.

Strain or plasmid	Relevant characteristics	Reference or source
***Escherichia coli* strains**		
DH5α	F^-^ *end A hsdR17* (rK^-^ mK^+^) *supE44 thi-1 recA1 gyr-A96* _*80lacZ*M15	Hanahan, 1983
MC1061	*thi thr1 leu6 proA2 his4 argE2 lacY1 galK2 ara14 xyl5, supE44*, aaa*pir*	Milton et al., 1996
***Aeromonas salmonicida* strains**		
A450	Wild type, subsp. *salmonicida*	Jimenez et al., 2009
CECT894	Wild type, subsp. *salmonicida*	CECT
CECT4235	Wild type, subsp. *salmonicida*	CECT
CECT896T	Wild type, subsp. *masoucida*	CECT
AS60	Wild type, subsp. *masoucida*	Austin et al., 1998
CECT4238	Wild type, subsp. *achromogenes*	CECT
CECT895T	Wild type, subsp. *achromogenes*	CECT
AS46	Wild type, subsp. *achromogenes*	Austin et al., 1998
AS102	Wild type, subsp. *achromogenes*	Austin et al., 1998
CECT5752T	Wild type, subsp. *pectinolytica*	CECT
CECT5753	Wild type, subsp. *pectinolytica*	CECT
CECT5179	Wild type, subsp. *smithia*	CECT
AS74	Wild type, subsp. *smithia*	Austin et al., 1998
A450ΔWasC	A450 *wasC* LPS-core in frame mutant	Jimenez et al., 2009
A450ΔWaaL	A450 *waaL* LPS-core in frame mutant	Jimenez et al., 2009
***A. hydrophila* strains**		
AH-3ΔwaaL	AH-3 *waaL* LPS-core in frame mutant	Jimenez et al., 2008
AH-3ΔwahD	AH-3 *wahD* LPS-core in frame mutant	Jimenez et al., 2008
**Plasmids**		
pGEMT easy	PCR generated DNA fragment cloning vector Amp^R^	Promega
pBAD33-Gm	Arabinose-inducible expression vector, Gm^R^	Jimenez et al., 2009
pBAD33-WasC_mas_	Vector with *wasC* from *A. salmonicida masoucida*	This study
pBAD33-WaaL_mas_	Vector with *waaL* from *A. salmonicida masoucida*	This study
pBAD33-WaaL_achr_	Vector with *waaL* from *A. salmonicida achromogenes*	This study
pBAD33-WaaL_pec_	Vector with *waaL* from *A. salmonicida pectinolytica*	This study
pBAD33-WaaL_smi_	Vector with *waaL* from *A. salmonicida smithia*	This study
pBAD33-WahD_pec_	Vector with *wahD* from *A. salmonicida pectinolytica*	This study

*^R^, resistant. CECT = SPANISH TYPE CULTURE COLLECTION.*

### Genetic General Methodology

General DNA manipulations were done essentially as previously described, as well as the DNA sequencing and bioinformatics analysis of sequenced data (Aquilini et al., 2014).

### Dot Blot Hybridizations

Total DNA was denatured after 5 min boiling, chilled on ice for 5 min. After, DNA samples were spotted onto prewetted in 2x SSC Hybond N1 (Amersham) nylon membrane and fixed by UV irradiation. Prehybridization was performed in a solution of 5x SSC, 0.1% *N*-lauroyl sarcosine, 0.02% SDS, 5% blocking reagent (Roche), and 50% formamide for 2 h at 42°C. Hybridization with the correspondent labeled probe (20 ng/ml) with digoxigenin was performed for 18 h at 42°C. The alkaline phosphatase detection system was finally carried out using the enhanced chemiluminescence detection system (Amersham) according to the manufacturer’s instructions.

### Plasmid Constructions for Gene Overexpression and Mutant Complementation Studies

For gene complementation studies of previously isolated *A. salmonicida* A450 and *A. hydrophila* AH-3 core mutants, the corresponding genes from chromosomal DNA of different *A. salmonicida* subspecies strains were PCR-amplified using specific primer pairs (**Table 2**) and ligated to pGEMTeasy plasmid. To generate pBAD33-Gm constructions pGEMT plasmids with the different genes were double digested with *Xba*I and *Sma*I and the DNA fragment obtained in each case ligated to pBAD33-Gm double digested with the same enzymes.

**Table 2:** Primers used for mutant complementation using vector pBAD33-Gm.

Plasmid	Primers	Amplified fragment (bp)
pBAD33-WasC_mas_	WasC-Mas For: 5′-tcc**CCCGGG**cagcgacgtaccatttgaa-3′	819
	WasC-Mas Rev: 5′-gcTCTAGAgaatccggtcgcgtaatag-3′	
pBAD33-WaaL_mas_	WaaL-Mas For: 5′-tcc**CCCGGG**gaagattcggggcaactac-3′	1333
	WaaL-Mas Rev: 5′-gcTCTAGAcaaggccaagatgcttcat-3′	
pBAD33-WaaL_achr_	WaaL-Acro For: 5′-tcc**CCCGGG**gaagattcggggcaactac-3′	1333
	WaaL-Acro Rev: 5′-gcTCTAGAcaaggccaagatgcttcat-3′	
pBAD33-WaaL_pec_	WbbB-FOR: 5′-tcc**CCCGGG**gaagattcggggcaactac-3′	1333
	WaaL-Pect Rev: 5′-gcTCTAGAcaatgccaagatgctccat-3′	
pBAD33-WahD_pec_	WahD-Pect FOR: 5′-tcc**CCCGGG**atcttcccaattcaacggc-3′	1281
	WahD-Pect Rev: 5′-gcTCTAGAcgacaagatcatcgccaat-3′	

*Primers contain SmaI(bold and capital letters) and XbaI(underlined and capital letters) restriction sites. The PCR amplified product was ligated to SmaI- XbaI digested pBAD33-Gm.*

Plasmid pBAD33-WaaL_smi_ was generated using the primers designed for pBAD33-WaaL_mas_. pBAD33-Gm plasmids into *E. coli* MC1061 were then transferred into the different mutants by triparental mating using the mobilizing strain HB101/pRK2073 (Jimenez et al., 2009). Mutants were selected on plates containing gentamicin and nalidixic acid for the A450strain, and gentamicin and rifampicin in case of the AH-3 strain. Each gene was expressed from the arabinose-inducible and glucose-repressible pBAD33-Gm promoter.

### LPS Isolation and SDS-PAGE

For screening purposes LPS was obtained after proteinase K digestion of whole cells and the LPS samples were separated by SDS-PAGE or SDS-Tricine-PAGE and visualized by silver staining as previously described (Aquilini et al., 2014). Cultures for analysis of LPS were grown in TSB at 20°C. Dried bacterial cells of each strain in 25 mM Tris⋅ HCl buffer containing 2 mM CaCl_2_ pH 7.63 (10 ml g^-1^) were treated at 37°C with RNAse, DNAse (24 h, 1 mg g^-1^ each), and then with proteinase K (36 h, 1 mg g^-1^). The suspension was dialyzed and lyophilized, and the LPS was extracted by the phenol-water procedure (Westphal and Jann, 1965). A portion of the LPS (∼50 mg) from each strain was heated with aqueous 2% acetic acid (6 ml) at 100°C for 45 min. The precipitate was removed by centrifugation (13,000*g* × 20 min) and the supernatant fractionated on a column (56 cm × 2.6 cm) of Sephadex G-50 (S) in 0.05 M pyridinium acetate buffer pH 4.5 with monitoring using a differential refractometer. An oligosaccharide fraction was obtained in a yield 9–20 % depending on the strain.

### Methylation Analysis and Mass Spectrometry

The methylation analyses were performed as previously described (Jimenez et al., 2009). Positive-ion reflectron time-of-flight mass spectra (MALDI-TOF) were acquired on a Voyager DE-PR instrument (Applied Biosystems) equipped with a delayed extraction ion source and used as previously described (Jimenez et al., 2009).

### Comparative Genomics and Reannotation

For each analyzed genome we gathered all CDS and pseudo-CDS information by parsing NCBI GenBank records. When we obtained the UniProt Knowledge Base records for these loci using the cross-reference with Entrez GeneIDs and parsed them for gene names, functional annotations, and associated COG, PFAM, and TIGRFAM protein domains were studied. To annotate orthologs, we wrote custom scripts to analyze reference sequence alignments made to subject genomes with blastn and tblastn via NCBI’s Web application programming interface. Briefly, we manually confirmed contextually accurate alignments, and then the script integrated coordinates and sequence information from both BLAST methods to locate the bounds of the reference gene in the subject genome; if an aligned start or stop codon was not located, we manually inspected the region. The script then analyzed alignments for insertions, deletions, premature stop codons, frameshifts, and changes to the start codon. An alignment in the same genomic context with >95% amino acid identity, excluding gaps and truncations, was our initial cutoff for orthology. The genomes of subsp. *salmonicida* A449, subsp. *masoucida* strain NBRC13784, subsp. *achromogenes* strain AS03 and subsp. *pectinolytica* strain 34melT are located at the GenBank accession numbers: CP000644, BAWQ00000000, AMQG00000000.2 and ARYZ00000000.2, respectively. The complete nucleotide sequences of the three *A. salmonicida* A450 chromosomal regions containing the LPS core biosynthetic genes described here have been assigned GenBank accession numbers FJ238464, FJ238465, and FJ238466, respectively. The complete nucleotide sequences of the three *A. hydrophila* AH-3 chromosomal regions containing LPS core biosynthesis genes described here have been assigned the following GenBank accession numbers: EU296246, EU296247, and EU296248.

## Results

**FIGURE 1:**
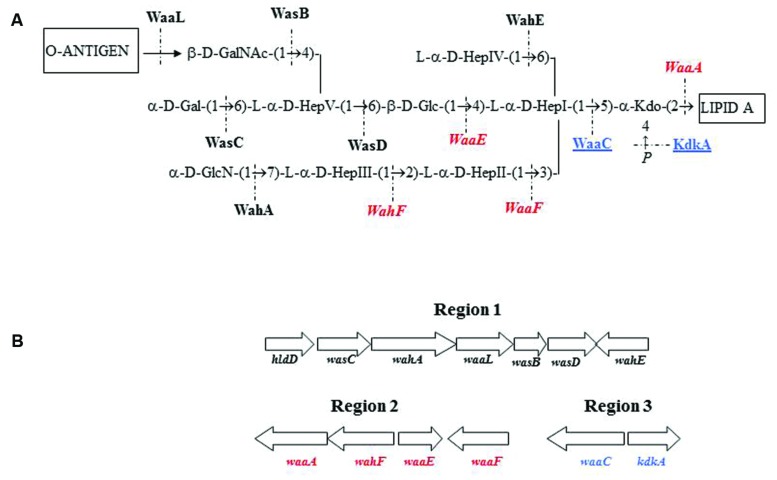
***Aeromonas salmonicida* subsp. *salmonicida* strain A450 chemical structure (A).** Proteins encoded by genes from different chromosomal regions are shown in roman type and black (region 1), in italics and red (region 2), or underlined and blue (region 3). **(B)** Genomic regions with the genes involved in the *A. salmonicida* subsp. *salmonicida* strain A450 *waa* (LPS-core biosynthesis).

We previously established the genomics and proteomics of the *A. salmonicida* subsp. *salmonicida* A450 strain *waa* (Jimenez et al., 2009; **Figure 1**). We studied by Colony Southern blot analysis, using several DNA probes, the *waa* region of *A. salmonicida* in subspecies *masoucida*, *achromogenes*, *pectinolytica*, and *smithia*. The initial selected DNA probes from strain A450 corresponded to complete *wasC* for chromosomal region 1, complete *waaE* for region 2, and complete *waaC* for region 3. WasC is the glycosyltransferase that links Gal to HepV in an α-1,6 linkage, WaaE the glycosyltransferase that links Glc to HepI in a β-1,4 linkage, and WaaC the heptosyltransferase that links HepI to Kdo in an α-1,5 linkage (**Figure 1**). A positive reaction was obtained with all the subspecies genomic DNA against probes from regions 2 and 3 (**Table 3**). However, either subspecies *pectinolytica* or *achromogenes* showed no reaction against *wasC* probe from region 1. Subspecies *masoucida* and *smithia* rendered a positive reaction against this probe.

**Table 3:** Hybridization studies with several DNA probes of *A. salmonicida* strains from different subspecies.

*A. salmonicida* strain	Hydridization with DNA probes		
	*wasC*	*waaE*	*waaC*
subsp. *salmonicida* A450	+	+	+
subsp. *salmonicida* CECT894	+	+	+
subsp. *salmonicida* CECT4235	+	+	+
subsp. *masoucida* CECT896T	+	+	+
subsp. *masoucida* AS60	+	+	+
subsp. *achromogenes* CECT4238	-	+	+
subsp. *achromogenes* CECT895T	-	+	+
subsp. *achromogenes* AS46	-	+	+
subsp. *achromogenes* AS 102	-	+	+
subsp. *pectinolytica* CECT5752T	-	+	+
subsp. *pectinolytica* CECT5753	-	+	+
subsp. *smithia* CECT5179	+	+	+
subsp. *smithia* AS74	+	+	+

*+, Positive reaction; -, negative reaction.*

When we used two additional DNA probes from region 1, *wahA* and *wasD* (**Figure 1**), a positive reaction was obtained with either subspecies *pectinolytica* or *achromogenes* genomic DNA. These results prompted us to study the LPS-core of the different *A. salmonicida* subspecies *masoucida*, *pectinolytica*, and *achromogenes*.

### *A. salmonicida* subsp. *masoucida*

**FIGURE 2:**
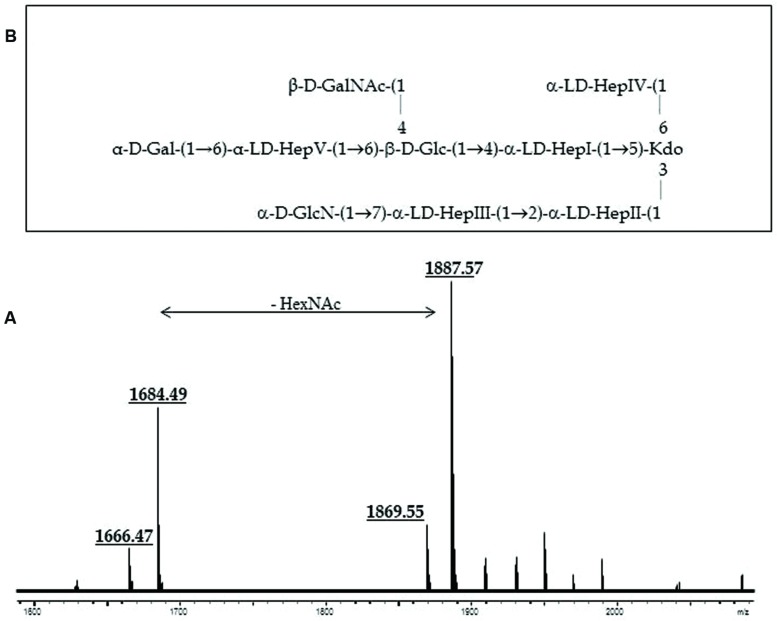
**(A)** Positive-ion MALDI-TOF of purified LPS core from *A. salmonicida* subspecies *masoucida* strain CECT8967T. **(B)** Presumptive LPS-core structure of the same strain according to the previously published *A. salmonicida* A450 (Jimenez et al., 2009).

Composition analysis of the strain CECT896T core oligosaccharide from purified LPS by GLC showed the presence of Glc, Gal, GlcN (glucosamine), GalNAc, L,D-Hep, and Kdo in the ratios 1:0.9:0.9:0.8:4.7:0.9, respectively. The mass spectrum from this core oligosaccharide sample showed a major molecular ion peak at *m/z* 1.888,60 (**Figure 2A**), corresponding to the full core (calculated molecular mass, 1.887,60 atomic mass units). This molecular mass is essentially similar to those reported for both wild-type *A. salmonicida* subsp. *salmonicida* strains A449 and 80204-1 (Wang et al., 2006). Similar to other reported cases, some structural heterogeneity was observed, which was associated with the existence of Kdo in both normal and anhydro forms. The signal observed could be attributed to Kdo_1_, Hep_5_, Hex_2_, HexN_1_, HexNAc_1_. Methylation analysis showed that the core oligosaccharide was characterized by containing similar molar ratios of terminal Gal, GlcN, GalNAc, and L,D-Hep. In addition, 6-substituted Glc, 2-substituted Hep, 7-substituted Hep, 4,6-bisubstituted Hep, and 3,4,6-trisubstituted Hep were found. The complete presumptive structure of the LPS from *A. salmonicida* subsp. *masoucida* strain CECT896T is shown in **Figure 2B**.

**FIGURE 3:**
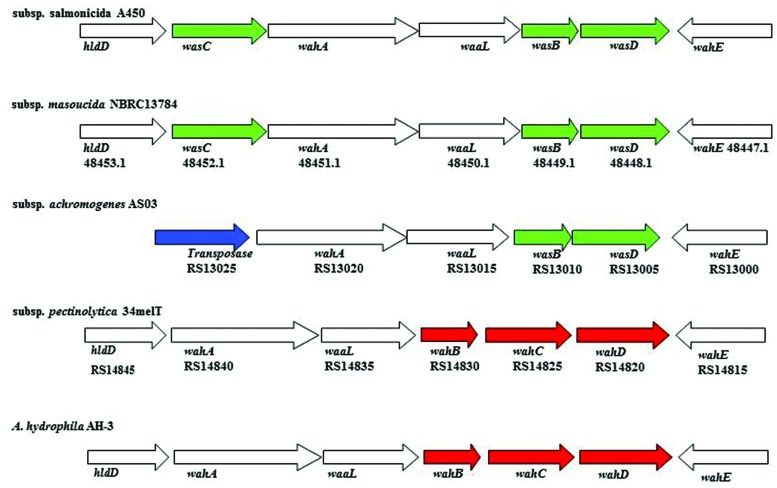
**The genes in region 1 of *A. salmonicida waa* from subspecies *salmonicida* A450; subspecies *masoucida* strain NBRC13784; subspecies *achromogenes* strain AS03; subspecies *pectinolytica* strain 34melT; and *A. hydrophila* strain AH-3.** The genes in green are unique for *A. salmonicida* strains, in red are initially unique for *A. hydrophila* strains, no color are shared by both species. The transposase is labeled in blue. The identity percentage of all the genes analyzed is over 97%.

**FIGURE 4:**
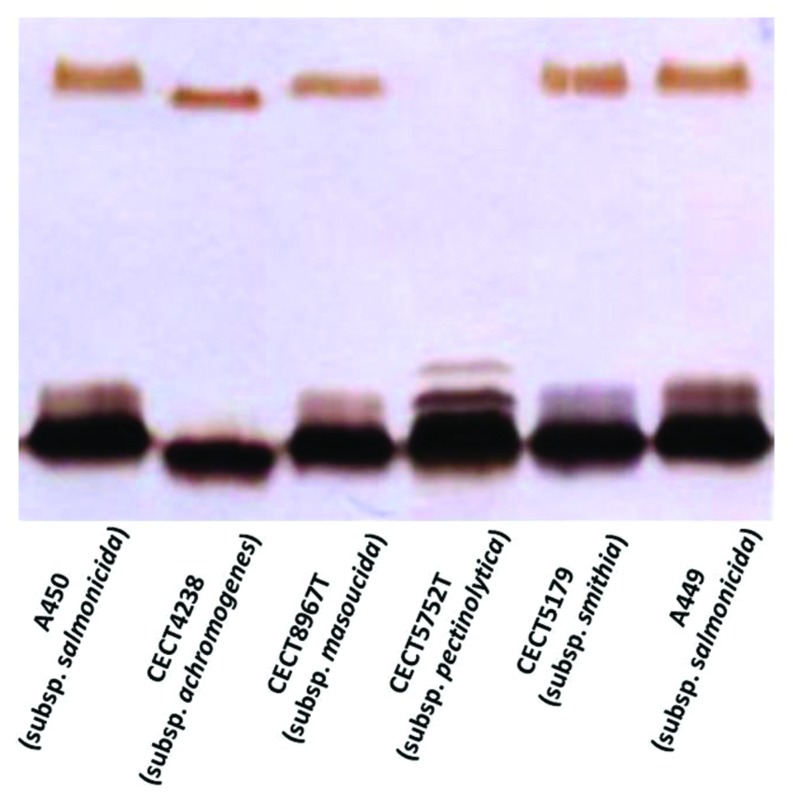
**Lipopolysaccharide (LPS) analyzed by SDS-Tricine gel and silver stained from the different subsp. of *A. salmonicida* strains**.

The annotation of the *waa* region in *A. salmonicida* subsp. *masoucida* strain NBRC13784 was revised. Comparative genome analysis between the reannotated and the ortholog region in *A. salmonicida* subsp. *salmonicida* strain A450 (Jimenez et al., 2009), showed identical genes (**Figure 3**). The predicted functions encoded by the reannotated *waa* gene cluster of this *A. salmonicida* subsp. *masoucida* were in agreement with the chemical data obtained. Furthermore, the relative mobility of the LPS-core in a silver-stained SDS-PAGE gel from *A. salmonicida* subsp. *masoucida* strain NBRC13784 was identical to the mobility of the LPS-core from strain *A. salmonicida* subspecies *salmonicida* strain A450 (**Figure 4**).

### *A. salmonicida* subsp. *achromogenes*

**FIGURE 5:**
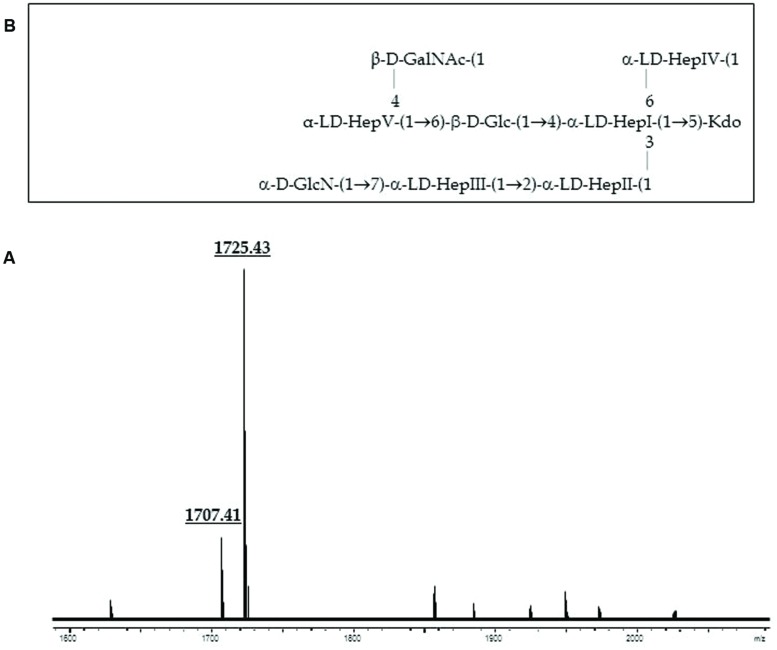
**(A)** Positive-ion MALDI-TOF of purified LPS core from *A. salmonicida* subspecies *achromogenes* strain CECT4238. **(B)** Presumptive LPS-core structure of the same strain according to the previously published *A. salmonicida* A450 (Jimenez et al., 2009).

GLC analysis of the strain CECT4238 core oligosaccharide from purified LPS showed the presence of Glc, GlcN, GalNAc, L,D-Hep, and Kdo in the ratios 1:1:0.9:4.5:0.9, respectively. The mass spectrum from this core oligosaccharide sample showed a major molecular ion peak at *m/z* 1.725,43 (**Figure 5A**), corresponding to the full core (calculated molecular mass, 1.726,10 atomic mass units). The signal observed was attributed to Kdo_1_, Hep_5_, Hex_1_, HexN_1_, HexNAc_1_. Similar to previous results some structural heterogeneity was observed due to Kdo in both normal and anhydro forms. Methylation analysis showed that the core oligosaccharide was characterized by containing similar molar ratios of terminal GlcN, GalNAc, and L,D-Hep. In addition, 6-substituted Glc, 2-substituted Hep, 7-substituted Hep, 4-substituted Hep, and 3,4,6-trisubstituted Hep were found. This core fraction was found to be essentially similar to those reported for wild-type *A. salmonicida* subsp. *salmonicida* strains, with the lack of the Gal linked in a α1-6 linkage to L,D-HepV (Jimenez et al., 2009). The complete presumptive structure of the LPS from *A. salmonicida achromogenes* is shown in **Figure 5B**. Only one complete genome of *A. salmonicida* subsp. *achromogenes* is currently available from strain AS03 (Han et al., 2013). When we revised this region by comparative genomics data in other *A. salmonicida*, we found the genes indicated in **Figure 3**, with a completely lack of *wasC* and *hldD* and the presence of a putative transposase. WasC is the glycosyltransferase that links Gal in an α-1,6 linkage to L,D-HepV in the LPS core of *A. salmonicida* subsp. *salmonicida* A450 (Jimenez et al., 2009; **Figure 1**) and HldD is the epimerase for the L,D-Hep and D,D-Hep (Read et al., 2004). The predicted functions encoded by the genes in this region were in agreement with the chemical data. Furthermore, the relative mobility of the LPS-core from *A. salmonicida* subsp. *achromogenes* strain CECT4238 is in a silver-stained SDS-PAGE gel was higher than the mobility of the LPS-core from strain *A. salmonicida* subsp. *salmonicida* strain A450 (**Figure 4**), which was in agreement with the loss of a monosaccharide residue (Jimenez et al., 2009).

### *A. salmonicida* subsp. *pectinolytica*

**FIGURE 6:**
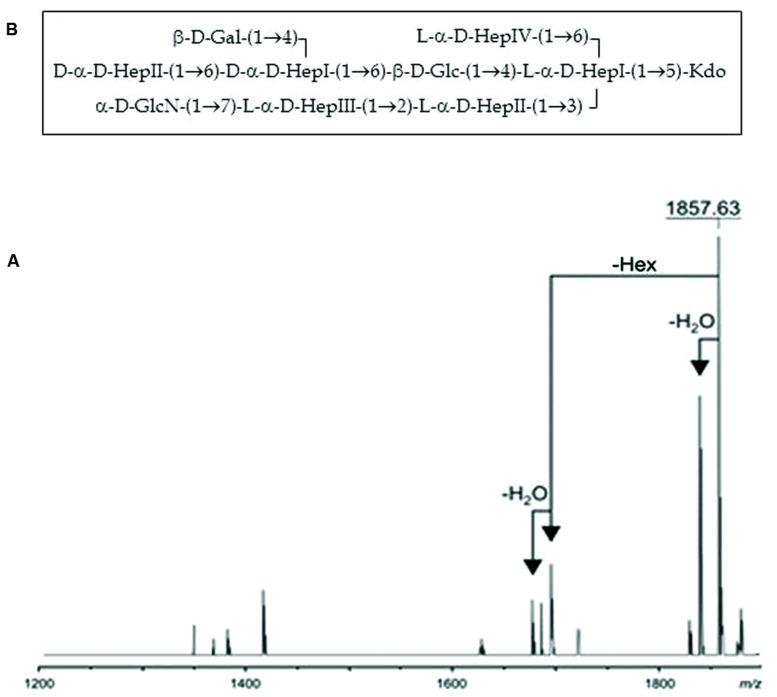
**(A)** Positive-ion MALDI-TOF of purified LPS core from *A. salmonicida* subspecies *pectinolytica* strain CECT5752T. **(B)** Presumptive LPS-core structure of the same strain.

Composition analysis of the strain CECT5752T core oligosaccharide from purified LPS by GLC revealed the presence of Glc, Gal, GlcN, D-glycero-D-manno-heptose (D,D-Hep), L-glycero-D-manno-heptose (L,D-Hep), and Kdo in the ratios 1:0.7:0.9:2.1:4.3, respectively. The major molecular ion peak at *m/z* 1.857,63 in its mass spectrum (**Figure 6A**) corresponded with calculated molecular mass 1.857,61 atomic mass units. The signal observed was attributed to Kdo_1_, Hep_6_, Hex_2_, HexN_1_. Methylation analysis resulted in identification of terminal Gal, 6-substituted Glc, terminal GlcN, terminal D,D-Hep, 6-substituted D,D-Hep, 4,6-disubstituted D,D-Hep, terminal L,D-Hep, 2-substituted L,D-Hep, 7-substituted L,D-Hep, and 3,4,6-trisubstituted L,D-Hep. The oligosaccharide sample from the *A. salmonicida* subsp. *pectinolytica* strain CECT5752T was found to be essentially identical to that of *A. hydrophila* AH-3 serogroup O34 (Jimenez et al., 2008), i.e., the same full core LPS. The complete presumptive structure of the LPS from *A. salmonicida pectinolytica* is shown in **Figure 6B**.

A comparative “in silico” analysis of the reannotated region 1 from the *A. salmonicida* subsp. *pectinolytica* strain 34melT showed identical genes to *A. hydrophila* AH-3 serotype O34 but not to any of the *A. salmonicida* strains. As can be observed in **Figure 3**, *A. salmonicida* subsp. *pectinolytica* strain 34melT shows *wahB*, *wahC*, and *wahD* genes from *A. hydrophila* AH-3 (in red) and lack the *wasB*, *wasC*, and *wasD* genes characteristic of *A. salmonicida* strains (in green). Also, **Figure 4** shows that this strain lacks the characteristic *A. salmonicida* O-antigen LPS and present some bands probably from another kind of O-antigen LPS (Merino et al., 2015).

### Complementation Studies

**FIGURE 7:**
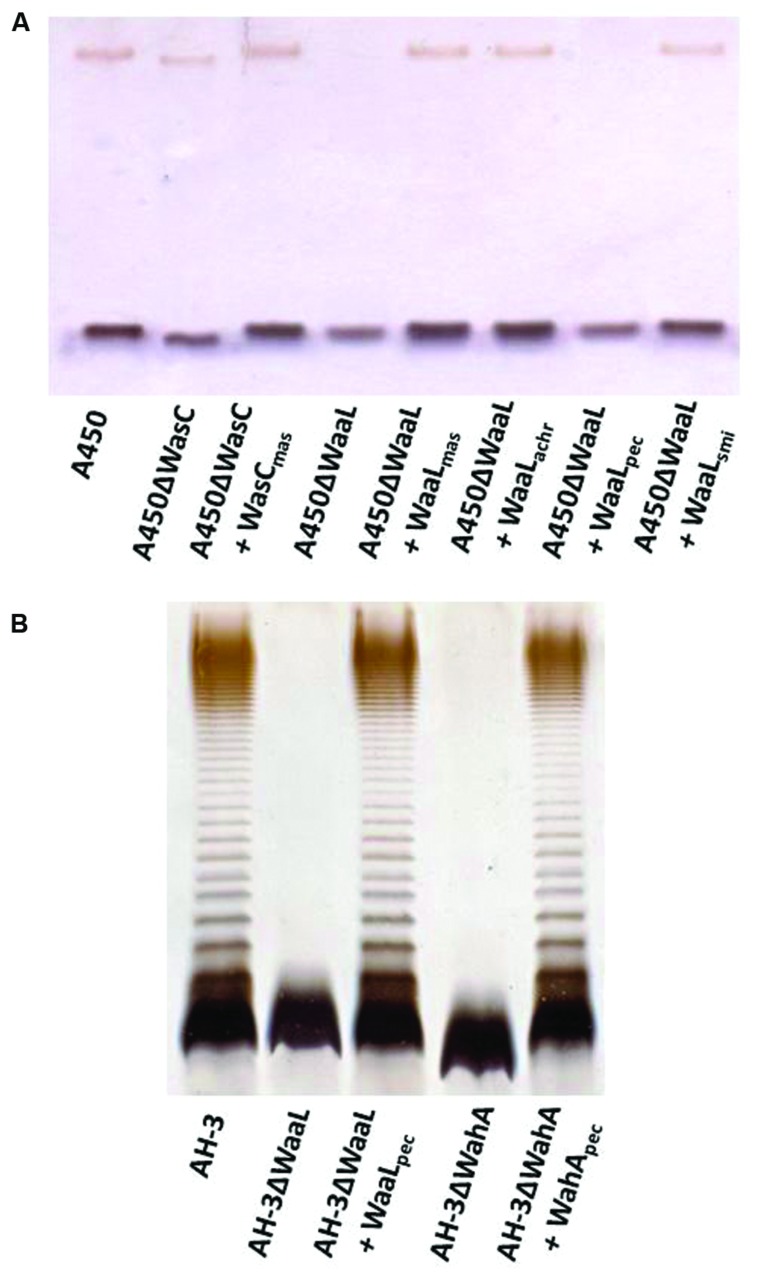
**(A)** Lipopolysaccharide analyzed by SDS-Tricine gel from *A. salmonicida* A450ΔWasC and A450ΔWaaL mutants and complemented mutant strains with pBAD33 plasmids carrying different single genes. **(B)** LPS analyzed by SDS-PAGE (12%) from *A. hydrophila* AH-3ΔWaaL and AH-3ΔWahA mutants and complemented mutant strains with pBAD33 plasmids carrying different single genes.

In order to confirm some of the gene identity, we initially study complementation in A450ΔWasC (formerly A450ΔORF2) and A450ΔWaaL mutants (Jimenez et al., 2009). As can be observed by SDS-PAGE or SDS-Tricine gels, *wasC* from *A. salmonicida masoucida* strain CECT896T (pBAD33-Gm-WasC) was fully able to complement A450ΔWasC mutant (identical migratory profile for LPS-core in gels, see **Figure 7A**). **Figure 7A** also showed that *waaL* from *A. salmonicida* subsp. *masoucida* strain CECT896T, from *A. salmonicida* subsp. *achromogenes* strain CECT4238, and from *A. salmonicida* subsp. *smithia* strain CECT5179 (pBAD33-Gm-WaaL_masoucida_, pBAD33-Gm-WaaL_achromogenes_, and pBAD33-Gm-WaaL_smithia_, respectively) were able to complement A450ΔWaaL mutant (recovery of the O-antigen LPS in gel). However, *waaL* from *A. salmonicida* subsp. *pectinolytica* strain CECT5752T (pBAD33-Gm-WaaL_pectinolytica_) was unable to do it (**Figure 7A**). Nevertheless, *waaL* from *A. salmonicida* subsp. *pectinolytica* strain CECT5752T (pBAD33-Gm-WaaL_pectinolytica_) was fully able to complement AH-3ΔWaaL mutant (Jimenez et al., 2008) judged by their LPS profile in gel (recovery of the O-antigen LPS bands), as shown in **Figure 7B**. Furthermore, *wahD* from *A. salmonicida* subsp. *pectinolytica* strain CECT5752T (pBAD33-Gm-WahD_pectinolytica_) was fully able to complement AH-3ΔWahD mutant (Jimenez et al., 2008) as can be judged by their LPS profile in gel **Figure 7B**, recovery of LPS-core mobility as the wild type strain and O-antigen LPS bands.

## Discussion

The bacterial species *A. salmonicida* comprises five subspecies. *A. salmonicida* subsp. *salmonicida* is known as typical *A. salmonicida*, causing furunculosis in salmonid fish (Bernoth, 1997). Atypical *A. salmonicida* include the other four subspecies: *masoucida*, *achromogenes*, *smithia*, and *pectinolytica* which, with the exception of *A. salmonicida* subsp. *pectinolytica*, are found as pathogens in a wide variety of fish species (Gudmundsdottir and Bjornsdottir, 2007). *A. salmonicida* subsp. *pectinolytica* strains are readily distinguished from the other psychrophilic aeromonads using the following phenotypic characteristics: growth at 35°C, melanin production, growth on KCN broth, mannitol and sucrose fermentation with gas from glucose, and indole plus Voges Proskauer assays. Its ability to degrade polypectate is an unusual feature among *Aeromonas* species (Pavan et al., 2000).

Interestingly, the structure of the LPS core oligosaccharide from *A. salmonicida* subsp. *pectinolytica* is also consistent with the established core structure of *A. hydrophila* strain AH-3 serotype O34 (Jimenez et al., 2008). Both structures are identical with respect to its inner and outer core regions with D-α-D-Hepp-(1→6)-[β-D-Galp-(1→4)]-D-α-D-Hepp-(1→) trisaccharide outer core fragment being present, while *A. salmonicida* subsp. *salmonicida* showed a trisaccharide outer core of α-D-Galp-(1→6)-β-D-GalpNAc-(1→4)-L-α-D-Hepp-(1→). When we inspected and deeply studied the LPS core gene clusters of *A. salmonicida* subsp. *pectinolytica* from the published fully sequenced genome, the predicted gene functions were in agreement with the chemical structure. Either by gene analysis or by complementation studies the region 1 of *waa* from *A. salmonicida* subsp. *pectinolytica* corresponds to the *A. hydrophila* AH-3 *waa* determined. The genomic analyses of the *A. salmonicida* subsp. *pectinolytica* region 1 from strain 34melT versus the *Aeromonas* whole genomes from mesophilic strains found in Pubmed (http://www.ncbi.nlm.nih.gov/genome/?term=Aeromonas) rendered that approximately 89% of the strains contain the same region 1. Nevertheless, from the 121 whole genomes inspected, 13 of them belonging to the species *A. hydrophila*, *A. veronii*, *A. caviae*, *A. media*, and *Aeromonas* sp. showed some different genes (**Table 4**).

**Table 4:** Genomic analyses of the *A. salmonicida* subsp. *pectinolytica waa* region 1 from strain 34melT versus the *Aeromonas* whole genomes from mesophilic strains.

Species	Number of Genomes		Percentage of genomes similar to *A. salmonicida* subsp. *pectinolytica*
	Analyzed	Similar to *A. salmonicida* subsp. *pectinolytica*	
*A. hydrophila*	39	35	89,7
*A. veronii*	29	27	93
*A. caviae*	11	8	72,7
*A. media*	1	0	0
*Aeromonas* sp.	6	3	50
*A. jandaei*	3	3	100
*A. schubertii*	2	2	100
*A. allosaccharophila*	3	3	100
*A. enteropelogenes*	4	4	100
*A. encheleia*	1	1	100
*A. popoffii*	1	1	100
*A. dhakensis*	4	4	100
*A. diversa*	2	2	100
*A. molluscorum*	1	1	100
*A. taiwanensis*	1	1	100
*A. lacus*	1	1	100
*A. finlandensis*	1	1	100
*A. aquatica*	1	1	100
*A. tecta*	1	1	100
*A. simiae*	1	1	100
*A. sanarelli*	1	1	100
*A. rivuli*	1	1	100
*A. piscícola*	1	1	100
*A. fluvialis*	1	1	100
*A. eucrenophila*	1	1	100
*A. bivalvum*	1	1	100
*A. australiensis*	1	1	100
*A. bestiarum*	1	1	100
	121	108	89

*Aeromonas salmonicida* subsp. *achromogenes* showed a disaccharide in its LPS outer core of β-D-GalpNAc-(1→4)-L-α-D-Hepp-(1→) instead of the previously mentioned *A. salmonicida* subsp. *salmonicida* trisaccharide. When we inspected and deeply studied the unique *A. salmonicida* subsp. *achromogenes* fully sequenced genome, the analysis and reannotation of the region 1 was in agreement with the biosynthesis of this chemical structure. The *wasC* and *hldD* were absent from region 1 of *A. salmonicida* subsp. *achromogenes waa* and instead a transposase was present. The transposase DDE found in subsp. *achromogenes* strain AS03 contains two domains Pfam 13737 and 01609, which are members of the DDE superfamily, which contain three carboxylate residues that are believed to be responsible for coordinating metal ions needed for catalysis. The catalytic activity of this enzyme involves DNA cleavage at a specific site followed by a strand transfer reaction. This family contains transposases for mainly insertion sequence (IS) 4 or 421 (Klaer et al., 1981). WasC is the glycosyl transferase that links Gal in a α1-6 linkage to L,D-HepV in the LPS core (**Figure 1**) and this monosaccharide residue is missing in the outer core LPS. By genomic analyses we could confirm the complete absence of *wasC* over the genome and only 126bp are retained between the transposase and *wahA* genes (11,2% of total gene). No fragment of *wasC*, was found retained upstream of the transposase gene. Therefore, a complex rearrangement event is probably responsible of the loss of the *hldD* and *wasC* genes. HldD (the epimerase for D,D-Hep) is not needed in *A. salmonicida* subsp. *achromogenes* LPS-core because D,D-Hep is not found. No *hldD* gene could be found by genomic analyses in the subsp. *achromogenes* strain AS03 total genome. Accordingly, the *A. salmonicida* subsp. *achromogenes* strains LPS-core migration in SDS-PAGE is faster than the one observed for LPS-core of *A. salmonicida* subsp. *salmonicida* strains.

No changes in the outer core trisaccharide (α-D-Galp-(1→6)-β-D-GalpNAc-(1→4)-L-α-D-Hepp-1→) are found in *A. salmonicida* subsp. *masoucida* strains, being region 1 of *A. salmonicida* subsp. *salmonicida waa* identical to the subspecies *masoucida* according to chemical structure data, genomic information, LPS-core SDS-PAGE gel migration, and complementation studies. Besides that no full genome is still available for *A. salmonicida* subsp. *smithia* strains, the complementation studies and the LPS-core SDS-PAGE gel migration suggest that region 1 of *A. salmonicida* subsp. *smithia* is probably identical to the one of *A. salmonicida* subsp. *salmonicida*.

No changes were observed in regions 2 and 3 of *waa* from *A. salmonicida* subspecies. These data were obtained either by hybridization analysis or by genome study of the different public complete genomes of *A. salmonicida* strains independently of the subspecies. Furthermore, the genomic analyses of the *Aeromonas* whole genomes from mesophilic strains found in Pubmed (http://www.ncbi.nlm.nih.gov/genome/?term=Aeromonas) indicate that these genomic regions were identical in all the *Aeromonas* strains studied, either psychrophilic or mesophilic.

**FIGURE 8:**
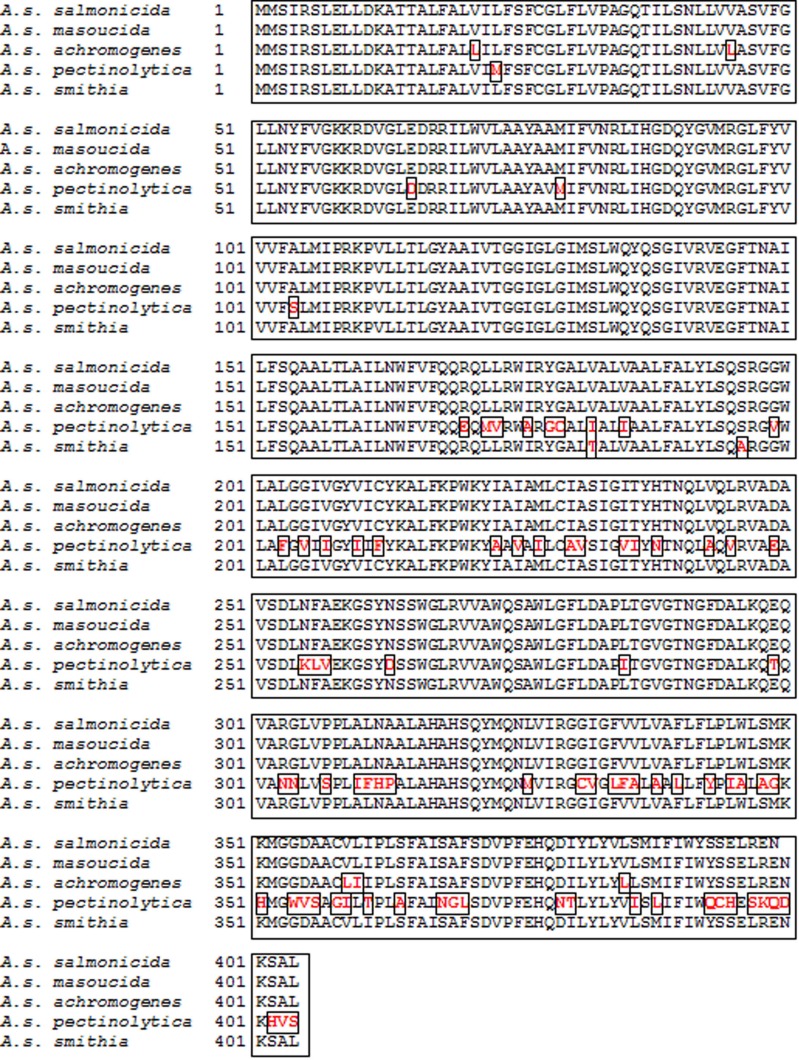
**Alignment of the WaaL aminoacid sequence from *A. salmonicida* subsp. *salmonicida* A450, *A. salmonicida* subsp. *masoucida* NBRC13784, *A. salmonicida* subsp. *achromogenes* strain AS03, *A. salmonicida* subsp. *pectinolytica* strain 34melT, and *A. salmonicida* subsp. *smithia* CECT5179.** Different aminoacids residues among the sequences are labeled in red and bold and inside a square box.

WaaL is the ligase enzyme that links the O-antigen LPS to the lipidA-LPS core, and shows two clear features. The enzyme catalyzes the formation of a glycosidic bond but does not share any protein motif with usual glycosyltransferases, and second the specificity of the reaction is based on the requirement for a specific lipid A-core OS acceptor structure but not the O-antigen LPS or any other undecaprenol-P-linked substrate (Valvano, 2011). According to these features, the WaaL from subsp. *salmonicida*, subsp. *masoucida*, and subsp. *smithia* are identical in amino acid sequence (**Figure 8**). WaaL subsp. *smithia* sequence was obtained after sequencing pBAD33-WaaL_smi_. WaaL from subsp. *achromogenes* showed a large similarity (nearly identity only with a few amino acid residues changes) to the previous ones, while WaaL from subsp. *pectinolytica* showed a clearly decreased similarity versus the rest of the WaaL from other *salmonicida* subspecies (**Figure 8**). The *A. salmonicida* subsp. *pectinolytica* WaaL from strain 34melT showed more identity with many WaaL from several mesophilic *Aeromonas* strains belonging to different species than to WaaL from other *A. salmonicida* subspecies.

It can be observed that the LPS inner core in *A. salmonicida* strains is well-conserved; however, there is some structural diversity in the LPS outer core. From the different typical and atypical *A. salmonicida* strains we can conclude that subsp. *salmonicida*, subsp. *masoucida*, and probably subsp. *smithia* strains shared the same kind of LPS outer core. *A. salmonicida* subsp. *achromogenes* strains showed a similar LPS outer core but lacked one branched external residue not affecting the O-antigen LPS linkage. However, *A. salmonicida* subsp. *pectinolytica* strains showed a rather changed LPS outer core, identical to many mesophilic *Aeromonas* strains LPS outer core. However, these LPS-core genes those are structural non-variable genes could be among others of interest for specific phylogenetic analyses.

## Author Contributions

All authors listed, have made substantial, direct and intellectual contribution to the work, and approved it for publication.

## Conflict of Interest Statement

The authors declare that the research was conducted in the absence of any commercial or financial relationships that could be construed as a potential conflict of interest.
